# Identification of immune-related biomarkers for glaucoma using gene expression profiling

**DOI:** 10.3389/fgene.2024.1366453

**Published:** 2024-04-17

**Authors:** Dangdang Wang, Yanyu Pu, Sisi Tan, Xiaochen Wang, Lihong Zeng, Junqin Lei, Xi Gao, Hong Li

**Affiliations:** ^1^ Department of Ophthalmology, The First Affiliated Hospital of Chongqing Medical University, Chongqing, China; ^2^ Chongqing Key Laboratory for the Prevention and Treatment of Major Blinding Eye Diseases, Chongqing Eye Institute, Chongqing Branch of National Clinical Research Center for Ocular Diseases, Chongqing, China

**Keywords:** biomarkers, immune-related genes, WGCNA, machine learning, immune cell infiltration, subclusters, glaucoma

## Abstract

**Introduction:** Glaucoma, a principal cause of irreversible vision loss, is characterized by intricate optic neuropathy involving significant immune mechanisms. This study seeks to elucidate the molecular and immune complexities of glaucoma, aiming to improve our understanding of its pathogenesis.

**Methods:** Gene expression profiles from glaucoma patients were analyzed to identify immune-related differentially expressed genes (DEGs). Techniques used were weighted gene co-expression network analysis (WGCNA) for network building, machine learning algorithms for biomarker identification, establishment of subclusters related to immune reactions, and single-sample gene set enrichment analysis (ssGSEA) to explore hub genes’ relationships with immune cell infiltration and immune pathway activation. Validation was performed using an NMDA-induced excitotoxicity model and RT-qPCR for hub gene expression measurement.

**Results:** The study identified 409 DEGs differentiating healthy individuals from glaucoma patients, highlighting the immune response’s significance in disease progression. Immune cell infiltration analysis revealed elevated levels of activated dendritic cells, natural killer cells, monocytes, and immature dendritic cells in glaucoma samples. Three hub genes, *CD40LG*, *TEK*, and *MDK*, were validated as potential diagnostic biomarkers for high-risk glaucoma patients, showing increased expression in the NMDA-induced excitotoxicity model.

**Discussion:** The findings propose the three identified immune-related genes (IRGs) as novel diagnostic markers for glaucoma, offering new insights into the disease's pathogenesis and potential therapeutic targets. The strong correlation between these IRGs and immune responses underscores the intricate role of immunity in glaucoma, suggesting a shift in the approach to its diagnosis and treatment.

## Introduction

Glaucoma, particularly primary open-angle glaucoma (POAG), represents a significant global health issue, identified as a leading cause of irreversible blindness. Characterized by degenerative optic neuropathy and the loss of retinal ganglion cells, POAG’s multifactorial etiology encompasses vascular, genetic, anatomical, and immune components (Allison et al., 2020). The prevalence of POAG is projected to escalate from approximately 57.5 million individuals globally to 76 million by 2020 and 111.8 million by 2040, underscoring its growing public health challenge (Allison et al., 2020).

The disease’s pathogenesis involves both innate and adaptive immune responses, indicative of glaucoma’s potential classification as an immune disorder. Early stages of glaucoma are marked by the activation of the eye’s innate immune cells, such as microglia, astrocytes, and Müller cells, which perform immune surveillance functions within the retina. These cells become activated, contributing to the disease’s progression. Systemic adaptive immune responses also play a critical role, with the detection of complex patterns of retinal proteins and autoantibodies against retinal specific antigens in the sera of glaucoma patients. Such immunological alterations, including the compromise of the blood-retina barrier and increased expression of matrix metalloproteinases, are pivotal in the disease’s development (Jiang et al., 2020; Baudouin et al., 2021). Additionally, neuroinflammation and oxidative stress are significant factors in POAG’s pathogenesis. The failure of the trabecular meshwork tissue in the conventional outflow pathway and the neuroinflammation process, which promotes neurodegeneration, are closely linked to age-related over-production of free radicals and oxidative stress-linked immunostimulatory signaling. Various oxidative stress-related markers have been identified in glaucoma patients, highlighting the complex interplay of cellular processes that promote POAG, including aging, oxidative stress, trabecular meshwork defects, glial cell activation, neurodegenerative insults, and altered regulation of immune response (Vernazza et al., 2020; Baudouin et al., 2021).

The main focus of traditional clinical approaches to glaucoma has been on evaluating and managing intraocular pressure (IOP). This involves conducting comprehensive ophthalmological exams, performing gonioscopy, and utilizing topical ocular antihypertensives, laser trabeculoplasty, or filtering surgery. While advancements in imaging technologies have improved glaucoma assessment, the primary objective of treatment remains reducing IOP (Kamat et al., 2016). Nevertheless, recent findings have started to unveil the intricate relationship between glaucoma and the immune system. Emerging evidence from studies involving animal models suggests that immune mechanisms play a significant role in the pathophysiology of the disease (Kamat et al., 2016). Furthermore, there is growing evidence indicating that the immune system significantly contributes to cell fate determinations in glia and retinal ganglion cells, thus contributing to the degeneration of the optic nerve in glaucoma. Immunoregulatory processes in glaucoma exhibit complex and sometimes contradictory roles. T cells that specifically target antigens can potentially shield neurons from the consequences of axonal injury, suggesting a neuroprotective aspect of the immune response. On the other hand, immune responses also have the potential to cause neuronal harm, highlighting the delicate balance between protective immunity and the risk of triggering autoimmune neurodegenerative processes (Tezel and Wax, 2004). Studies have demonstrated that prolonged inflammatory processes in the retina and optic nerve of glaucoma patients can lead to the production of neurotoxic mediators. However, our understanding of the precise molecular responses and interactions between different immune cells that determine the neuroinflammatory phenotype and its role in neurodegenerative outcomes is still limited (Bariş and Tezel, 2019).

In general, disorders related to immune regulation tend to be linked with diseases due to the wide range of functions that the immune system has in various physiological processes. The immune system plays vital roles in the development of glaucoma, including autoimmune degeneration of neurons, changes in immune responses, activation of microglial cells in the retina, presence of a pro-inflammatory environment, modulation of T cells, loss of retinal ganglion cells mediated by antibodies, immune response induced by stress, and maintenance of immune regulatory balance. Nevertheless, there is a lack of a comprehensive understanding regarding the relationship between the immune system and glaucoma, and the research focusing on the detailed functions of the immune system in glaucoma is limited. To investigate the potential pathogenesis of glaucoma, we conducted an analysis of the differences in gene expression between samples from healthy individuals (controls) and glaucoma patients using the Expression Omnibus (GEO) database. Through the utilization of machine learning techniques, we were able to identify a signature composed of immune-related genes. Based on this signature, we categorized glaucoma patients into two distinct groups with significantly different patterns, and examined the variances in immune cell populations and functions between these subgroups. The expression of hub genes was experimentally validated by constructing a cell model of glaucoma. This analysis aims to provide a new perspective on the molecular mechanisms underlying the development of glaucoma, as well as to contribute to the improvement of diagnostic, prognostic, and therapeutic strategies for this disease.

## Materials and methods

### Datasets collection

The datasets GSE2378 and GSE9944 were obtained and downloaded from the Gene Expression Omnibus (GEO) database available at https://www.ncbi.nlm.nih.gov/geo/. GSE9944 selected data from the GPL8300 platform with a large number of samples, including 13 glaucoma samples and six normal samples. GSE2378 selected the data measured by the GPL8300 platform with a large number of samples, including seven glaucoma samples and six normal samples. The Import database (https://www.immport.org/shared/home) was utilized to acquire the Immune-Related Genes (IRGs) for this study. To integrate the transcriptomic data from the aforementioned datasets, we initially employed the “Combat” function implemented in the “sva” R package (Chen et al., 2011; Leek et al., 2012) to remove any batch effects. The ComBat method was employed to standardize the expression values across different batches or platforms. Furthermore, Principal Component Analysis (PCA) was performed to assess the effectiveness of the batch effect removal process.

### Identification and visualization of differentially expressed genes (DEGs)

The analysis of differentially expressed genes (DEGs) was conducted through the utilization of the Limma package in the R programming language (Ritchie et al., 2015). Following quantile normalization, the raw data underwent logarithmic transformation. DEGs were determined based on specific criteria, including |logFC| > 0.5 and an adjusted *p*-value <0.05. To visualize the DEGs, specific R packages were employed, namely, pheatmap, dplyr, ggplot2, and ggrepel. Heatmaps and volcano plots were generated to represent the DEGs accordingly.

### WGCNA

To begin with, the R WGCNA software package was utilized for the computation of gene pair correlation coefficients via Pearson’s correlation coefficient, thereby constructing the gene co-expression matrix. Following the principle of a scale-free network, soft thresholds (power = 20) were chosen sequentially in order to establish a scale-free co-expression network. Consequently, the adjacency matrix was transformed into a topological overlap matrix. Subsequently, cluster analysis was executed to determine gene modules, ensuring that each module consisted of at least 60 genes. By means of hierarchical clustering, a dendrogram was formulated to assess the correlation between the characteristic genes of the modules and the disease phenotype. Ultimately, the module exhibiting the highest correlation coefficient and the smallest *p*-value was identified as the disease-specific module. By imposing a gene significance (GS) > 0.5 threshold and a module membership (MM) > 0.8 criterion, the core genes within the module were selectively filtered. Notably, the salmon module demonstrated the strongest correlation, with a maximum correlation value of 0.71 and an exceedingly small *p*-value of 5 × 10^−6^.

### Machine learning

The identification of immune-related DEGs was accomplished by intersecting DEGs, key module genes from the WGCNA screen, and genes that are linked to the immune system. Two machine learning techniques, namely, the least absolute shrinkage and selection operator (LASSO) (Tibshirani, 1996) and random forest (RF) (Ishwaran and Kogalur, 2010), were utilized to filter the potential POAG genes. To execute this task, the R packages “glmnet” and “randomForest” were employed. The LASSO regression technique was employed to enhance the accuracy and comprehensibility of the model through regularization. Additionally, LASSO regression enables the selection of relevant variables (Tibshirani, 1996). On the other hand, when it comes to predicting continuous variables while minimizing variance, RF emerges as a suitable technique due to its ability to achieve high precision, sensitivity, and specificity. Furthermore, RF does not impose any restrictions on the conditions of variables (Ishwaran and Kogalur, 2010). Using the aforementioned methods, the three most critical hub genes associated with the immune system were identified in this study.

### Gene function enrichment analysis

We utilized the R package (clusterProfiler, enrichplot) to carry out an analysis of Gene Ontology (GO) (http://geneontology.org/, accessed on 14 June 2023) and Kyoto Encyclopedia of Genes and Genomes (KEGG) (http://www.kegg.jp/or http://www.genome.jp/kegg/, accessed on 20 June 2023). This analysis focused on the enrichment of intersecting genes, which consisted of DEGs, key module genes identified through the WGCNA screen, and immune-related genes. A significantly different level for the enrichment result was determined at a *p*-value of less than 0.05 (Ogata et al., 1999).

### ssGSEA

The ssGSEA enrichment analysis was conducted by utilizing the GSVA package in R. In the initial step, the expression matrix of the patient samples in the POAG group was utilized to compute the percentage of immune cell infiltration and the activity score of immune-related pathways for each sample. To assess the disparities in the proportion of immune cell infiltration and the activity of immune-related pathways between the POAG group and healthy controls, a comparison was carried out using the vioplot package in R. In the subsequent phase, the R packages (ggplot2 and reshape2) were employed to establish a correlation between the expression levels of hub genes in the POAG group and the proportion of immune cell infiltration as well as immune-related pathways. The comparison was based on Spearman’s rank correlation measure. The final visualization is presented using heatmaps and box plots.

### Differential expression analysis and receiver operating characteristic (ROC) curve validation

In order to analyze and compare the expression levels of the POAG group and the control group in terms of the final hub genes, we utilized the R (limma and ggpubr) packages. The obtained data was then visualized through the implementation of boxplots. Additionally, for each hub gene, ROC curve analysis was conducted using the R pROC package (Robin et al., 2011), and subsequently, the area under the curve (AUC) was calculated. By considering the AUC value, we were able to determine the significance of the immune response, with values approaching one indicating a greater accuracy in model training.

### Nomogram construction and verification

To demonstrate the associations between the three key genes related to immune occurrence in POAG, an illustrative nomogram was developed with the assistance of the “rms” package in the R software (Shariat et al., 2009). The scores of a potential gene were indicated by “points”, while the sum of all gene scores was represented by “Total Points”. By employing the calibration curve and C-index, the performance of the nomogram was evaluated, visually representing the alignment between predicted probabilities and observed probabilities. This evaluation underscored the crucial significance of immune response in POAG.

### Unsupervised clustering of three immune-related genes

Using the “ConsensusClusterPlus” R package, we performed a consensus cluster analysis. This analysis utilized mRNA expression data from three genes associated with the immune system. For the CC parameter, we selected a maximum value of 3. To determine clusters, we employed the pam function as the ClusterAlg and utilized the Euclidean distance metric.

### Prediction of potential drugs

The DGIdb database (https://www.dgidb.org/search interactions) was employed to anticipate potential drugs for treating glaucoma based on the biomarkers associated with this condition. Utilizing Cytoscape software (version 3.10.1), the visual representation of the biomarker-compound pair network was generated.

### Cell culture

Mouse retinal ganglion (RGC-5) cell line (iCell Bioscience Inc., Shanghai, China) was cultured in Dulbecco’s modified Eagle’s Medium Nutrient Mixture F12 (DMEM-F12, GIBCO, Invitrogen, MA, USA) medium containing 10% Gibco fetal bovine serum (FBS, GIBCO, Invitrogen, CA, USA), and 1% penicillin/streptomycin (Procell, Wuhan, China) in a humidified atmosphere with 5% CO2 at 37°C. Excitotoxicity culture conditions were achieved with 2.5 mM *N*-methyl-d-aspartic acid (NMDA) (MCE, Shanghai, China) for 8 h (Li et al., 2018a; Li et al., 2018b).

### Real-time PCR

Total RNA was extracted from cells and tissues using the Trizol Reagent (Invitrogen, CA, USA). PrimeScript RT reagent Kit (MCE, Shanghai, China) was used to generate cDNA. Real-time PCR was performed with a system (ABI Prism 7500, Applied Biosystems, CA, USA) by using iTaq universal SYBR Green Supermix (BIO-RAD, CA, USA). The primer sequences are listed in the Supplementary Table S2. Relative mRNA expression was calculated with the 2^−ΔΔct^ method.

### Statistical analysis

All statistical analyses were performed in R language (version 4.3.1). All statistical tests were bilateral (*p* < 0.05).

## Results

### DEGs screening

**FIGURE 1:**
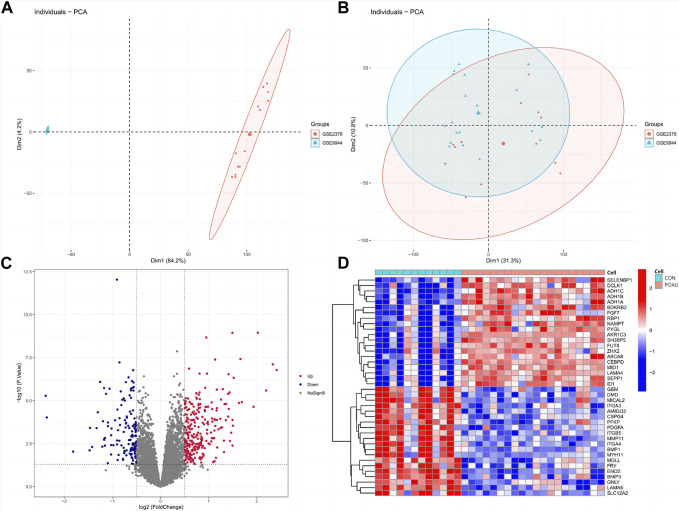
DEGs between POAG and CON samples. **(A,B)** Principal component analysis (PCA) of the GSE2378 and GSE9944 dataset. The points of the scatter diagrams diaplay the samples based on the top two principal components (PC1 and PC2) of gene expression profiles without **(A)** and with **(B)** the removal of batch effect. The dots in the graph represent samples, and the colors represent corresponding samples different datasets. **(C,D)** Volcano plot showing DEGs in POAG patients versus healthy controls **(C)** and the heatmap of TOP40 differential genes (upregulated and downregulated, **(D)**. POAG, primary open-angle glaucoma; CON, healthy controls.

In order to eliminate the batch effect between the GSE2378 and GSE9944 datasets, we implemented the ComBat approach. Figures 1A, B showcased the results before and after standardization. Initially, the samples from both datasets were grouped based on the top two principal components (PCs) of the expression values that had not been normalized, revealing the presence of the batch effect (Figure 1A). However, after performing cross-platform normalization, the scatter-plot depicting the PCA of the normalized expression level clearly demonstrated the successful removal of the batch effect arising from different platforms (Figure 1B). These findings indicated that the cross-platform normalization effectively eliminated the batch effect. Ultimately, we obtained normalized expression data from 20 samples of POAG and 12 healthy samples. Furthermore, employing criteria such as |logFC| > 0.5 and adjusted *p* < 0.05, a total of 409 DEGs were identified in our study using the normalized expression data. Among these DEGs, 267 genes were found to be upregulated while 142 genes were downregulated (Figure 1C). To visually represent these findings, we selected the top 40 genes from the upregulated and downregulated genes respectively, and created a heatmap in which red denoted upregulated genes and blue denoted downregulated genes (Figure 1D).

### Construction of co-expression network and identification of POAG core genes

**FIGURE 2:**
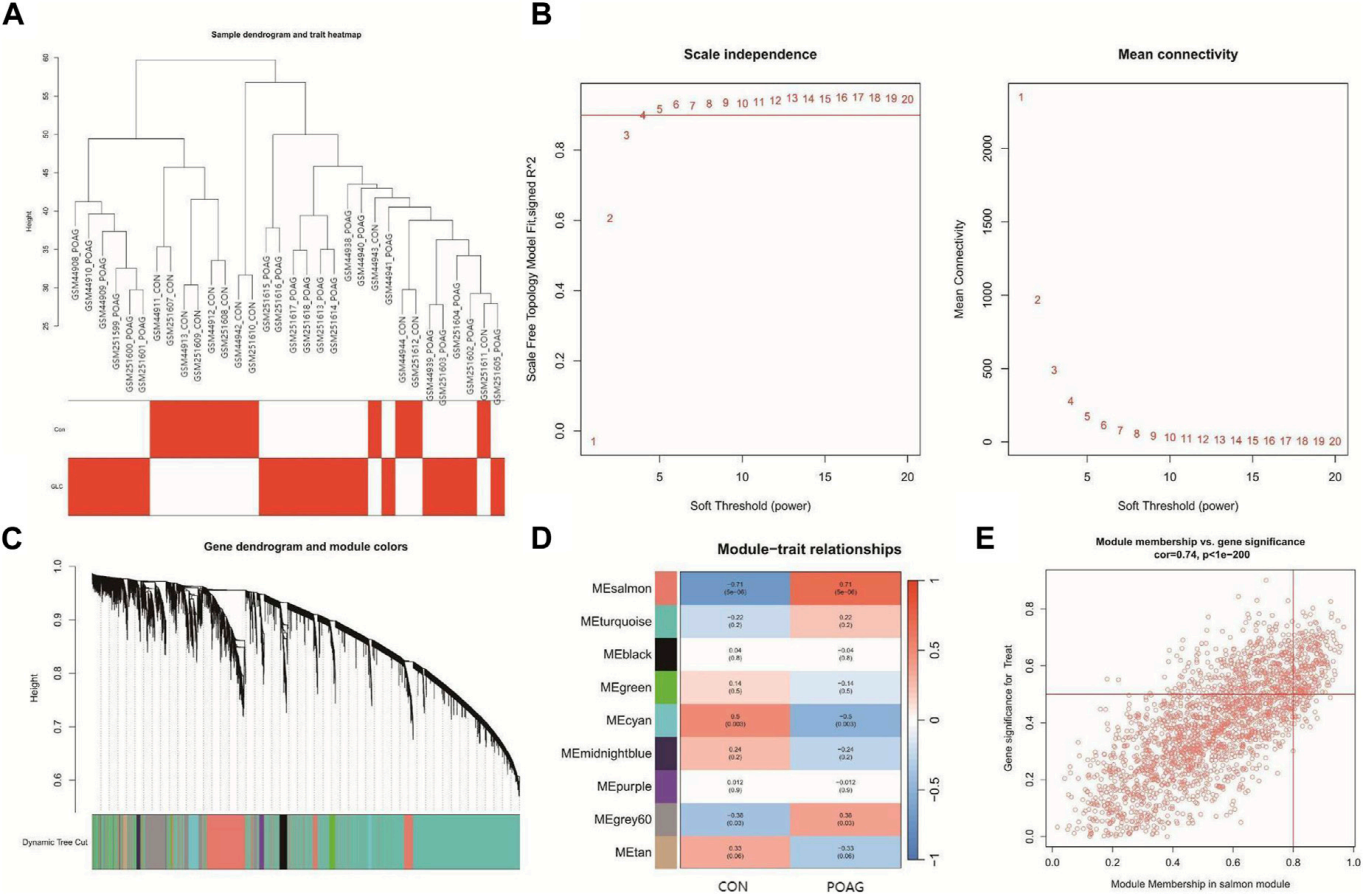
Construction of the co-expression network. **(A)** The sample dendrogram and feature heat map were drawn based on the Euclidean distance using the average clustering method for hierarchical clustering of samples, with each branch representing a sample, Height in the vertical coordinate being the clustering distance, and the horizontal coordinate being the clinical grouping information. **(B)** Soft threshold (power = 20). **(C)** Gene hierarchy tree-clustering diagram. The graph indicates different genes horizontally and the uncorrelatedness between genes vertically, the lower the branch, the less uncorrelated the genes within the branch, i.e., the stronger the correlation. **(D)** Heatmap showing the relations between the module and POAG features. The values in the small cells of the graph represent the two-calculated correlation values cor coefficients between the eigenvalues of each trait and each module as well as the corresponding statistically significant *p*-values. Color corresponds to the size of the correlation; the darker the red, the more positive the correlation; the darker the blue, the more negative the correlation. **(E)** Scatter plot between gene significance (GS) and module membership (MM) in salmon.

In order to more accurately discover the central genes linked to the POAG phenotype, a gene co-expression network was constructed using the WGCNA algorithm. The results of the hierarchical cluster analysis for the samples demonstrated well-defined clusters with no significant outliers (Figure 2A). To achieve a scale-free topology for the network, a soft threshold of 20 was applied (Figure 2B). By correlating genes, a dendrogram for gene hierarchy clustering was generated, and a total of nine modules with similar gene patterns were identified (Figure 2C). Ultimately, the module called “salmon” was determined to be the most clinically significant module in relation to POAG, as it contained 2038 genes and exhibited a correlation of 0.71 with a *p*-value of 5 × 10^−6^ (Figure 2D). The scatter plot (Figure 2E) illustrates a strong correlation of 0.74, with a *p*-value less than 1 × 10^−200^, between GS and MM within the “salmon” module.

### Selection and functional enrichment analysis of immune-related genes in POAG

**FIGURE 3:**
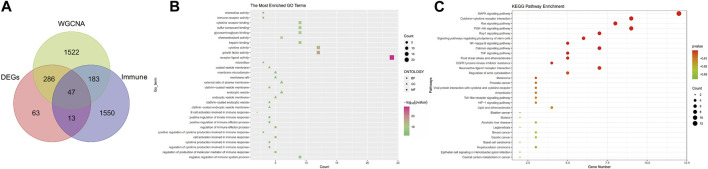
Screening for Immune-related signature genes in POAG and functional enrichment analysis. **(A)** Venn diagram of the intersection of DEGs, salmon module genes, and Immune-related genes. **(B)** GO functional annotation of signature genes. **(C)** Functional annotation of the Kegg signaling pathway of signature genes. For all enriched GO and KEGG terms, *p* < 0.05.

To explore the regulatory role of immune in the pathogenesis of POAG, we first intersected DEGs, salmon module genes, and immune-related genes to obtain a total of 47 signature genes (Figure 3A). Subsequently, we performed functional enrichment analysis of these signature genes to explore the biological functions and potential pathways associated with immune that occur in POAG. The results of GO enrichment analysis showed that the signature genes mainly affect host inflammation (endocytic vesicle membrane, endocytic vesicle, clathrin−coated endocytic vesicle), immune regulation (negative regulation of immune system process, positive regulation of cytokine production involved in immune response, B cell activation), cytokine activity and immune receptor activity (Figure 3B). The results of the KEGG signaling pathway analysis demonstrated that the signature genes were most enriched in inflammatory pathways (e.g., NF-κB, TNF, Toll−like receptor, cytokine receptors, etc.); immune-related pathways (Toll−like receptor, cytokine receptors, HIF-1, NF-κB, PI3K−Akt, Ras, MAPK); microbial infection and intestinal homeostasis (HIF-1, *Helicobacter pylori* infection, Legionellosis, Malaria, Amoebiasis); poor blood circulation (Fluid shear stress, atherosclerosis, Lipid, Calcium) (Figure 3C). These are in line with the inflammatory “storm”, microbiota disruption, and poor blood circulation that cause POAG, and they indirectly point to the immune disorder as a potential major component in the pathogenesis of pro-inflammatory immunity during POAG.

### Identification of the hub genes most associated with immune occurrence in POAG

**FIGURE 4:**
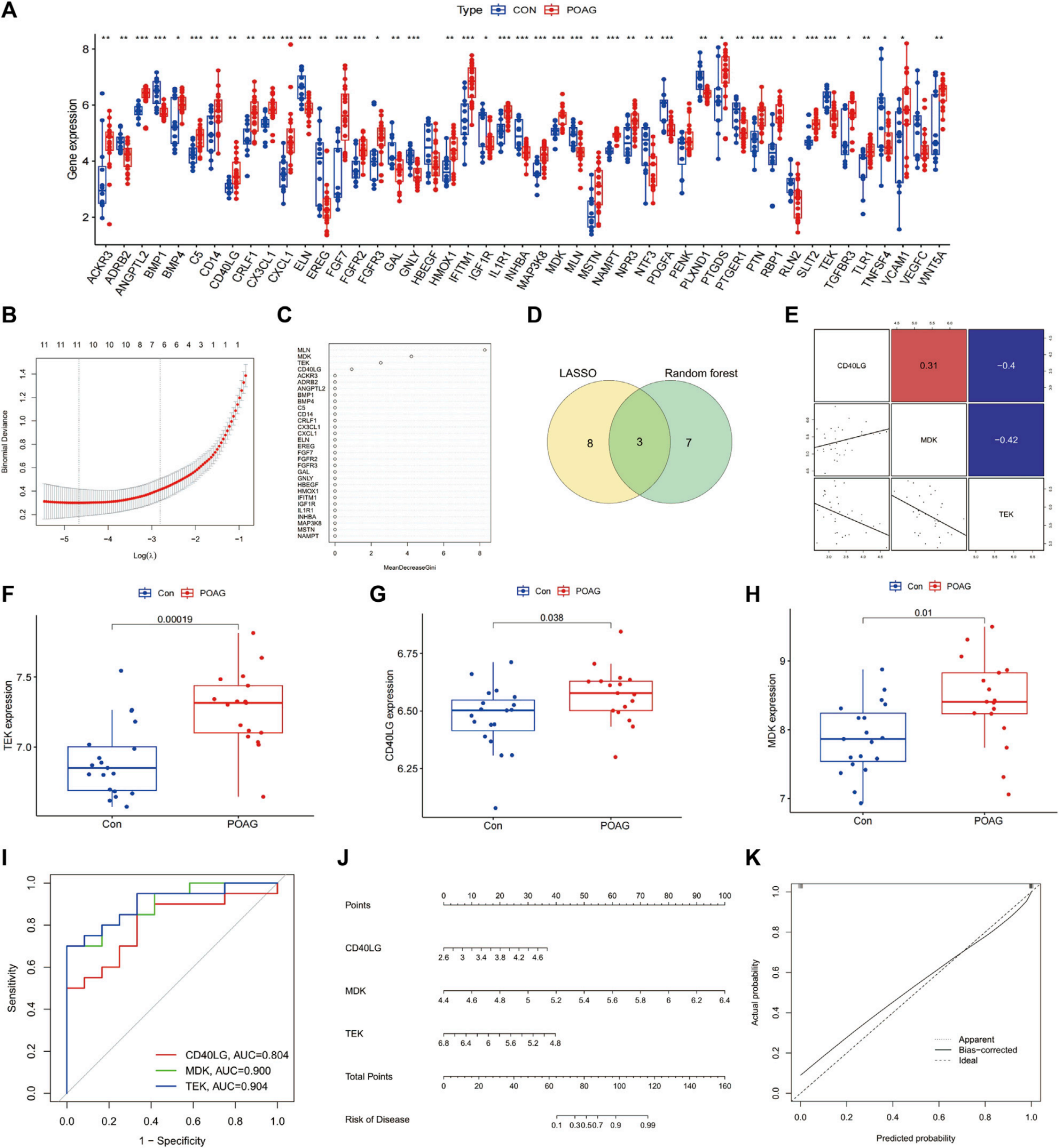
Screening of the Immune-related gene signature. **(A)** The expression of immune-related DEGs. **(B,C)** Construction of immune-related gene signature using LASSO and RF. **(D)** The overlap of genes in two machine learning. **(E)** Pairs plot showing the relationship between the immune-related gene signature. **(F–H)** Expression levels of three hub genes in POAG patients compared with healthy controls, *p < 0.05; ***p < 0.001. **(I)** ROC curve of immune-related hub genes in POAG diagnosis. DEGs, differentially expressed genes; LASSO, least absolute shrinkage and selection operator; RF, random forest; ROC, receiver operating characteristic. *p < 0.05; ***p < 0.001. **(J)** Nomogram for measuring the significance of immune in POAG based on hub genes. **(K)** Calibration curve plot for the nomogram. The *X*-axis represents the predictable probability, and the *Y*-axis represents the actual probability. Perfect prediction corresponds to the ideal dashed line. The apparent dashed line represents the entire queue, bias-corrected solid line is bias-corrected by bootstrapping (1,000 repetitions) and represents the observed performance of the nomogram. POAG, primary open-angle glaucoma; CON, healthy controls.

Three immune-related hub genes, namely, *CD40LG*, *MDK,* and *TEK*, were identified by LASSO and RF machine learning techniques based on the expression of 47 immune-related DEGs. The analysis of Figures 4A–E revealed the correlation between these hub genes. In POAG patients, the expression of *CD40LG, MDK*, and *TEK* was found to be upregulated compared to healthy controls (all *p* < 0.05), as shown in Figures 4F–H. By analyzing the AUC values of the ROC curve, it was determined that these three hub genes exhibited high sensitivity and specificity in assessing the importance of immune response in POAG (Figure 4I). To evaluate the clinical applicability of the binomial regression model, a nomogram depicting the hub genes was constructed (Figure 4J). The calibration plots in Figure 4K demonstrated a strong fit between the predicted and actual probabilities, supporting the presence of immune response in POAG.

### Immune cell infiltration/functions and its association with hub genes

**FIGURE 5:**
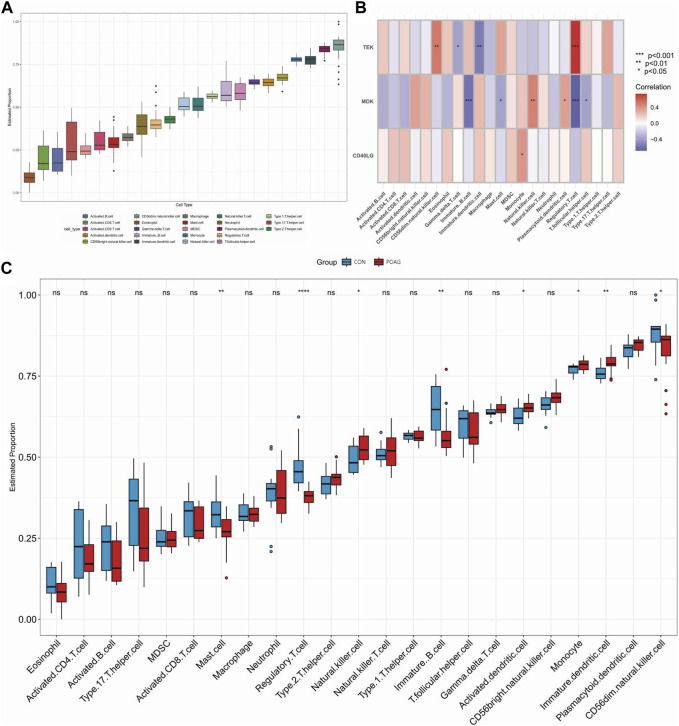
Analysis of immune infiltration in POAG patient samples and its correlation with hub genes using ssGSEA. **(A)** Box plot showing the immune proportion of 23 immune cells. **(B)** Correlation between immune cell infiltration and three hub genes. **(C)** Grouped box plot showing immune proportion of 23 immune cells in POAG patients and healthy controls. *p < 0.05, **p < 0.01, ***p < 0.001. POAG, primary open-angle glaucoma; CON, healthy controls.

**FIGURE 6:**
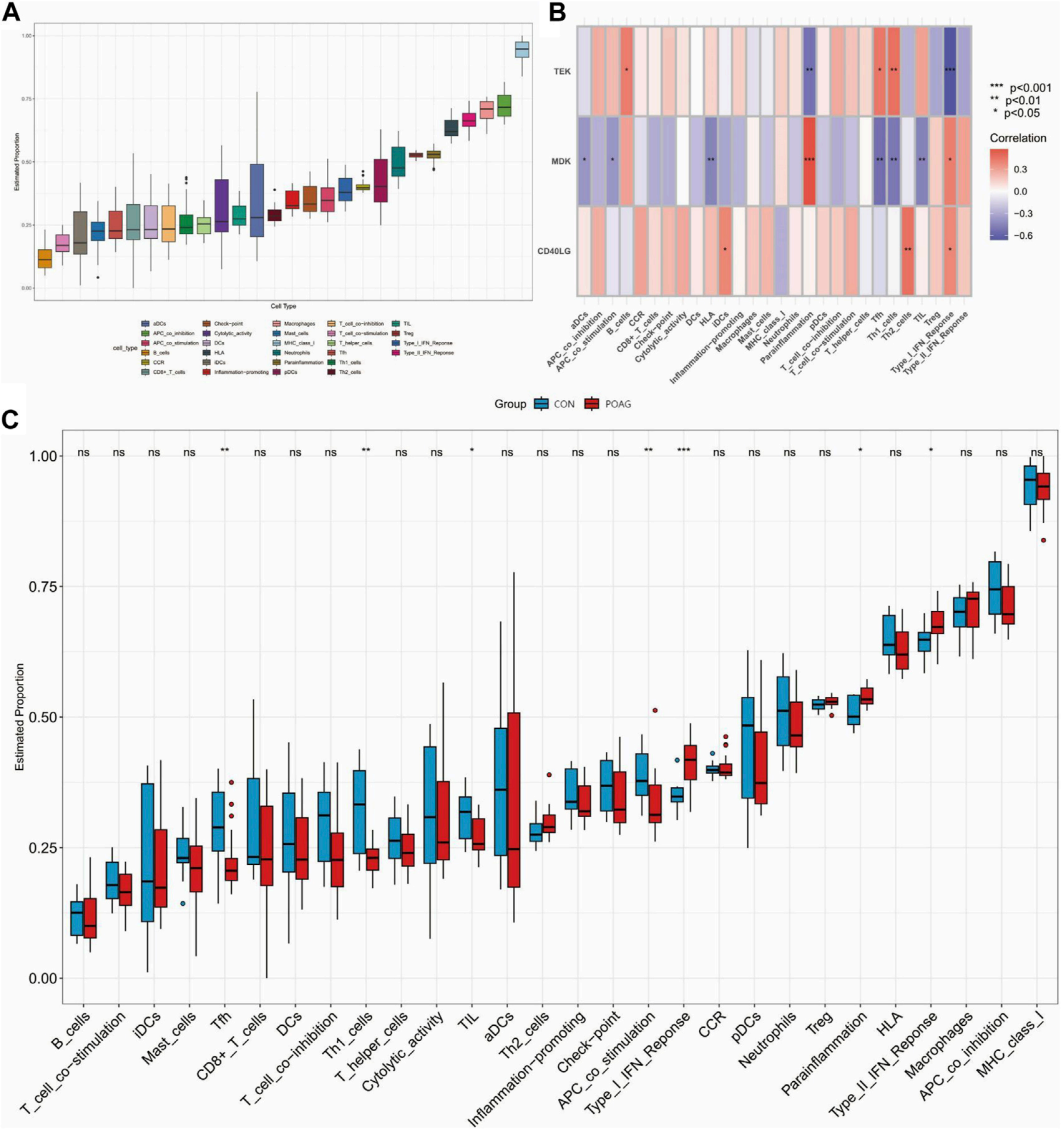
Analysis of immune functions in POAG patient samples and their correlation with hub genes using ssGSEA. **(A)** Box plot showing immune proportion for 10 immune functions in POAG patients and healthy controls. **(B)** Association between immune cell function and three hub genes. **(C)** Grouped box plot showing immune proportion for 10 immune functions in POAG patients and healthy controls; *p < 0.05, **p < 0.01, ***p < 0.001. POAG, primary open-angle glaucoma; CON, healthy controls.

Differences in the expression of immune infiltrate between POAG patients and healthy controls were further investigated using ssGSEA. Figures 5A, 6A show the distribution of 23 immune cells and 10 immune functions in the normalized expression data, respectively. In the box plot, the horizontal axis represents the different types of immune cells or immune-related functions, while the vertical axis shows the estimated proportion of each cell type or function within the samples. Consistency in the box sizes and median lines across samples of the same group suggests uniformity in the data distribution, while significant variations might indicate heterogeneity in cellular composition or immune function activity within the group. We observed a significantly higher infiltration of activated dendritic cell, natural killer cell, monocyte and immature dendritic cell and lower infiltration of immature B cell, mast cell, regulatory T cell and CD56dim natural killer cell in samples from POAG patients, respectively, suggesting a key role for these cells in the progression of POAG (Figure 5C). Correlation analysis showed that *TEK* was positively correlated with CD56dim natural killer cell and regulatory T cell and negatively correlated with gamma delta T cell and immature dendritic cell (all *p* < 0.05) (Figure 5B). In contrast, *MDK* was significantly positively correlated with natural killer cell and plasmacytoid dendritic cell and significantly negatively correlated with immature B cell, mast cell, regulatory T cell and T follicular helper cell. However, *CD40LG* was only significantly positively correlated with monocyte (all *p* < 0.05). These results simultaneously provide further evidence for the cellular–molecular mechanics by which immune plays a regulatory role in the progression of POAG. Furthermore, differential analysis of immune function showed that APC co-stimulation, parainflammation and type I IFN response were closely associated with immune in POAG (Figures 6B, C).

### Key gene-related pathways

In this analysis, we employed a combination of three crucial genes as the gene set to delve deeper into the transcriptional regulatory network pertaining to these essential genes. To predict the relevant transcription factors, we utilized the Cistrome DB online database. Notably, our predictions yielded 11 transcription factors through *MDK*, six transcription factors through *TEK*, and nine transcription factors through *CD40LG*. To visually represent the findings, we constructed a comprehensive transcriptional regulatory network for the key immune-related genes using Cytoscape (Supplementary Figure S1).

**FIGURE 7:**
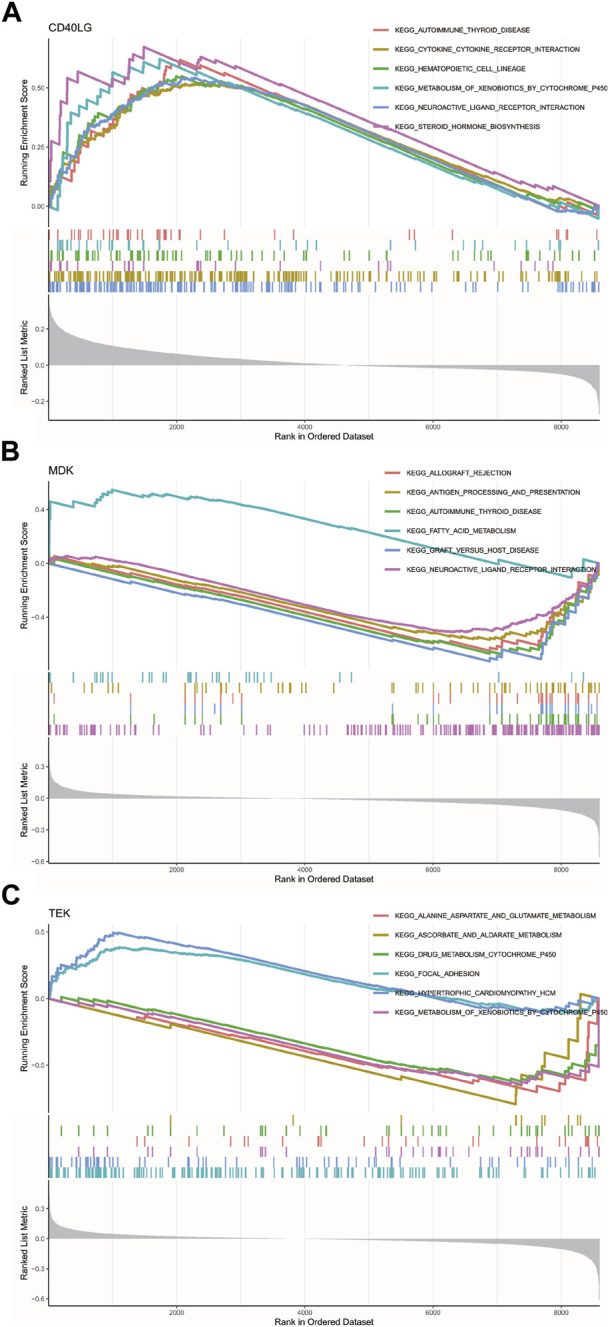
GSEA of KEGG enrichment analysis for the key genes. **(A)** GSEA of KEGG enrichment analysis for *CD40LG*. **(B)** GSEA of KEGG enrichment analysis for *MDK*. **(C)** GSEA of KEGG enrichment analyses for *TEK*. GSEA, gene set enrichment analysis. CD40 Ligand, *CD40LG*; Midkine, *MDK*; and *TEK* Receptor Tyrosine Kinase, *TEK*.

We studied the specific signaling pathways enriched by three key genes to explore the potential molecular mechanism of key genes affecting the progression of POAG. We selected the significantly enriched pathways shown in Figure 7. The pathways enriched with *CD40LG* by KEGG included immunomodulatory responses of cytokines and their receptor interactions, cytochrome P450-mediated metabolic processes and the biosynthesis of steroid hormones (Zhang et al., 2019). The pathways enriched with *MDK* by KEGG analysis included immunomodulatory responses in antigen-related processes, fatty acid-mediated metabolic processes and nervous system functional regulation. The pathways enriched with *TEK* by KEGG analysis included drug metabolism reactions, liver metabolism, cell adhesion and cardiac diseases (Figure 7).

### Diagnostic genes based ceRNA network

In order to identify potential miRNAs, an analysis was conducted on the three crucial genes using the miRWalk database. Initially, we extracted the mRNA-miRNA relationship pairs associated with these three key mRNAs from the miRWalk database. After applying filters, only 71 mRNA-miRNA relationship pairs meeting the criteria of TargetScan one or miRDB one were retained (comprising three mRNAs and 71 miRNAs). Ultimately, the construction of the ceRNA network was performed utilizing Cytoscape (V3.10.1) (Supplementary Figure S2).

### Immune-related gene signature-based consensus clustering analyses

**FIGURE 8:**
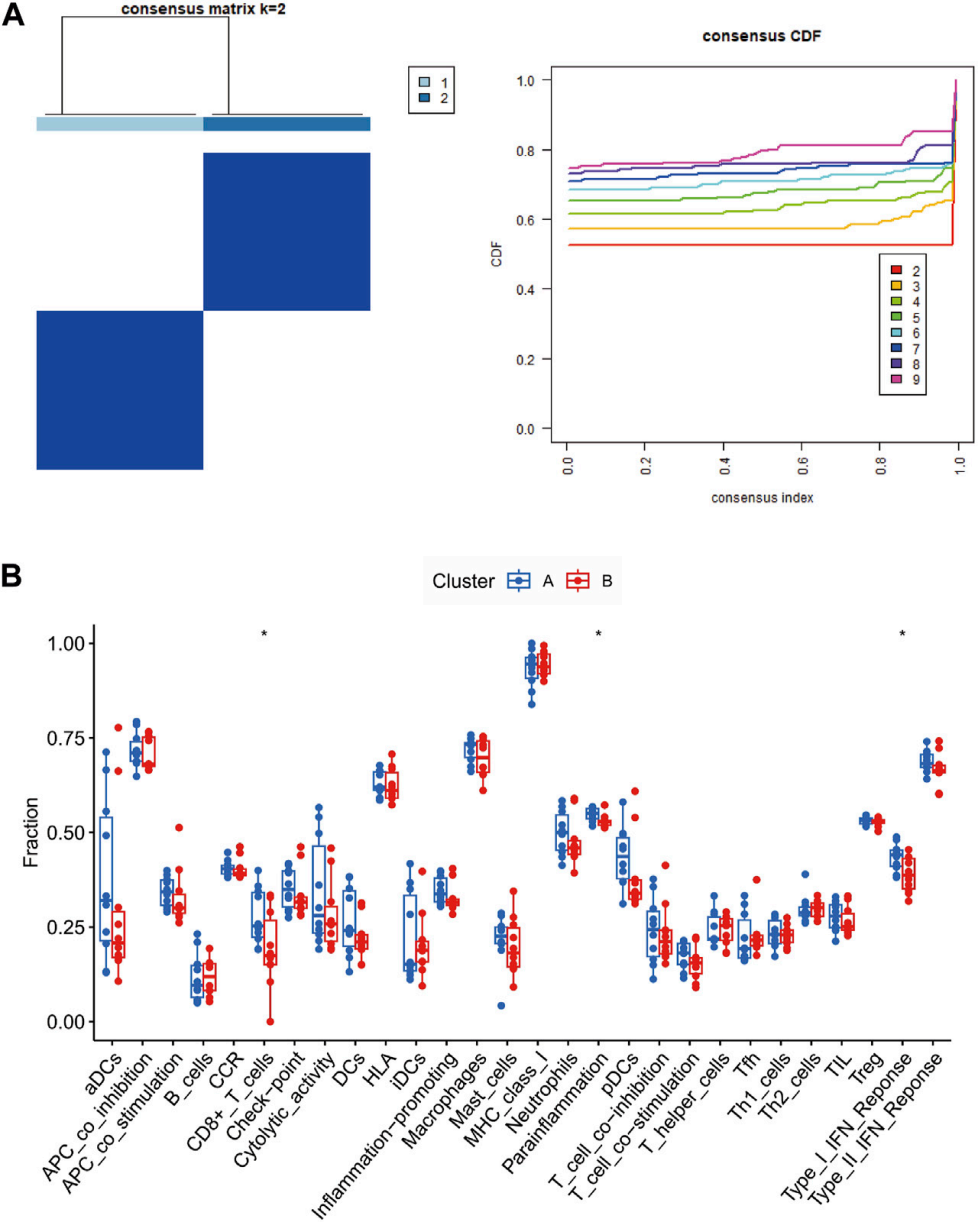
Identification of immune-related subtypes in glaucoma. Subclusters identified with the immune-related hub genes in dataset **(A)**. Boxplot **(B)** showing the correlation between 18 immune cells and 10 immune functions in subtypes. CDF, cumulative distribution function. Cluster A, POAG with high risk; Cluster B, POAG with low risk.

Consensus clustering was conducted on these three central genes related to the immune system, aiming to detect fresh subcategories of POAG patients. Within POAG samples, when k-value two was applied, it successfully divided them into two distinctive clusters, thereby pointing out significant variations in gene expression between these two groups (Figure 8A). It can be inferred from our findings that the POAG high-risk cluster (designated as cluster A) demonstrated elevated expression levels of CD8+T cell, parainflammation, and Type I IFN response (Figure 8B). All of these results collectively imply that immune-related pathways in the high-risk cluster (cluster A) were enhanced and relatively different from those observed in the low-risk cluster (cluster B).

### Potential drugs targeting the diagnostic genes

To investigate potential therapeutic drugs for glaucoma, we conducted a search in the DGIdb database for drugs that target specific biomarkers. As depicted in Supplementary Figure S3, we identified seventeen drugs that target *TEK* and eleven drugs that target *CD40LG*. Previous clinical trials have demonstrated the efficacy of one particular medication from the aforementioned list in treating open angle glaucoma or ocular hypertension (Brigell et al., 2022). In animal studies, three medications have exhibited anti-fibrotic properties and neuroprotective effects (Nemoto et al., 2019; Zhang et al., 2019; Kim et al., 2021). Additionally, two medications have been found to interfere with the TGF-β/p38 signaling pathway, modify cellular structure, and reduce extracellular matrix (ECM) at the cellular level (Luo et al., 2014; Song et al., 2022) (Supplementary Table S3).

### Clinical and cell validation of hub genes

To validate the expression of three genes in glaucoma, RT-qPCR was conducted. The findings demonstrated a significant elevation in mRNA expressions of *CD40LG*, *MDK*, and *TEK* in model groups when compared to the control groups (Supplementary Figure S4). Clinical data showed that patients with glaucoma in the high-risk group present with impaired eye function and inflammatory cell infiltration, mainly in the form of visual field defect, optic atrophy, thinner RNFL thickness, higher intraocular pressure, worse best-corrected visual acuity (BCVA), lower blood pressure, higher lymphocyte count and lower neutrophil cell count (Supplementary Table S1).

## Discussion

Glaucomas encompass a range of conditions leading to irreversible vision loss, characterized by the gradual decline of retinal ganglion cells (RGCs) and optic nerve damage, often associated with elevated intraocular pressure (Quigley, 1999). Our clinical findings indicated that high-risk group glaucoma presented more severe ocular structural and functional impairments compared to the low-risk group, including increased intraocular pressure, reduced best-corrected visual acuity (BCVA), visual field defects, optic atrophy, and thinner RNFL thickness. Interestingly, our analysis suggested a tendency towards higher lymphocyte count and lower neutrophil cell count in high-risk group glaucoma versus low-risk group (Supplementary Table S1). However, our comprehension of the molecular factors contributing to poor outcomes in glaucoma patients was currently limited. Glaucoma affected an estimated 3.54% of the global population, impacting approximately 79 million individuals and causing visual impairment and daily activity disruptions for many more (Tham et al., 2014). Presently, there were no available treatments to restore lost vision from glaucoma, emphasizing the importance of early detection and effective intervention (Jayaram et al., 2023). While lowering intraocular pressure had benefited numerous glaucoma patients, its clinical efficacy for an increasing number of individuals remained inadequate (Jayaram et al., 2023; Traverso et al., 2023). Thus, understanding the molecular mechanisms driving glaucoma development and devising novel therapies based on these mechanisms were crucial for enhancing clinical outcomes.

The field of immune research had rapidly advanced in recent decades, evolving from a focus on basic biology to a crucial area of clinical relevance. Researchers were now exploring the potential of utilizing the immune system for disease diagnosis and treatment (Stephen and Hajjar, 2020). Immune factors had also shown promise as diagnostic tools for glaucoma, shifting from traditional retinal-based methods to more accessible biological fluids like blood and aqueous humor (Von Thun Und Hohenstein-Blaul et al., 2017). Our study represented the first comprehensive analysis of three distinct immune patterns in POAG samples, identifying two unique immune clusters. We identified 409 DEGs between HC and POAG patients, with GO and KEGG enrichments highlighting the critical role of immune response in POAG development and progression. Analysis revealed a higher presence of activated dendritic cells, natural killer cells, monocytes, and immature dendritic cells in POAG samples, in line with prior studies (Forrester et al., 2010; Howell et al., 2012; Mastropasqua et al., 2016; Williams et al., 2019; Yu et al., 2020; Zhang et al., 2022). Research had shown enhanced NK cell activation in trabecular meshwork tissue of POAG patients and the detrimental effects of proinflammatory monocytes infiltrating the optic nerve in glaucoma models (Howell et al., 2012; Zhang et al., 2022). A study by Williams et al. (2019) demonstrated that inhibiting monocytes from entering the optic nerve could offer neuroprotection in cases of glaucoma. Furthermore, research had shown a higher density of dendritic cells in glaucoma groups compared to controls, indicating a possible involvement in the development of glaucoma-related ocular surface disease (Forrester et al., 2010; Mastropasqua et al., 2016). These findings collectively supported the significant role of immunity in the progression of glaucoma.

The immune system plays a crucial role in the development and functioning of biological processes, with immune dysfunction being closely linked to various diseases. In the context of glaucoma, hosts and the immune system have been extensively studied. Elevated intraocular pressure (IOP) triggers secondary immune responses that contribute to retinal ganglion cell (RGC) degeneration in glaucoma. The immune system has been explored as a potential source of biomarkers for glaucoma (Tezel and Wax, 2004; Vu et al., 2012; Kamat et al., 2016; Jiang et al., 2020). However, the significance of immune-related genes (IRGs) in glaucoma has not been definitively established, warranting further investigation. Utilizing two machine learning algorithms, three hub genes (*CD40LG*, *TEK*, and *MDK*) were identified. Subsequent analysis was conducted to determine the correlation between these genes and glaucoma. It was observed that IRGs interacted either synergistically or antagonistically with glaucoma patients. Moreover, the hub IRGs were validated for identifying high-risk groups for glaucoma, demonstrating effective discrimination. The research did not directly find a connection between *CD40LG* and glaucoma or its clinical implications for treatment. However, studies on *BACH2’s* role in immune response regulation and T-cell lymphoblastic leukemia progression emphasized the importance of understanding molecular pathways involving *CD40LG* for potential therapeutic targets (Feng et al., 2024). While specific studies on glaucoma were not identified, exploring *TEK* mutations, especially in primary congenital glaucoma (PCG), sheds light on genetic contributions to ocular diseases. Recent research has revealed the significance of *TEK*, a gene encoding Tie2 receptor tyrosine kinase, in Schlemm’s canal development and function, a crucial component of the eye’s aqueous humor outflow pathway. Variants in *TEK* have been linked to PCG, indicating a substantial genetic basis for this type of glaucoma. A Mendelian randomization study suggested a potential association between *TEK* signaling pathways and elevated intraocular pressure (IOP), a primary risk factor for glaucoma. By utilizing genetic instruments and single-nucleus RNA sequencing data, this study proposed a biological mechanism through which *TEK* may impact glaucoma pathogenesis (Rajasundaram et al., 2023). The intricate genetic landscape of Primary Congenital Glaucoma (PCG) was further complicated by interactions between *TEK* and *CYP1B1* genes, hinting at a digenic mode of inheritance in specific cases. These interactions may disrupt normal ocular development, leading to elevated Intraocular Pressure (IOP) and glaucoma. Ongoing research aimed to unravel the specific mechanisms by which these interactions influence the disease process, shedding light on PCG pathogenesis and the potential for targeted therapies (Kabra et al., 2017). Functional analysis of *TEK* mutations in Chinese PCG patients underscored the gene’s importance in disease development. Next-generation sequencing has revealed both loss-of-function and potentially activating mutations in *TEK*, providing valuable insights into its role in ocular health and disease. These findings emphasized the importance of comprehensive genetic screening in glaucoma patients and suggested personalized therapeutic strategies targeting the *TEK* pathway (Qiao et al., 2021). In summary, understanding *TEK’s* involvement in glaucoma through genetic, molecular, and functional studies held promise for clinical applications. Insights into *TEK’s* impact on ocular disease mechanisms may pave the way for innovative diagnostic and therapeutic approaches, offering hope for enhanced care of glaucoma patients. Midkine (*MDK*) plays a role in glial activity, neuronal survival, and the reprogramming of Müller glia into Müller glia-derived progenitor cells (MGPCs) (Campbell et al., 2021). While most literature focuses on *MDK’s* role in cancer progression and its potential as a therapeutic target due to its involvement in cell growth, survival, metastasis, migration, and angiogenesis in various cancers (Filippou et al., 2020). The inquiry about *MDK* and its potential clinical translation or treatment significance for glaucoma appeared to be an under-explored area in the highlighted articles.

Using unsupervised cluster analysis, two clusters associated with immune-related genes (IRGs) were identified based on three hub genes. Furthermore, immune cell infiltration analysis was conducted within these subgroups. Interestingly, the high-risk group A showed a higher proportion of adaptive immune cells, specifically CD8^+^ T cells. This may be attributed to an autoimmune or immune response triggered by elevated intraocular pressure (IOP) and the targeting of antigens such as heat shock proteins (HSPs) by T cells (Chen et al., 2018). The shift in T-cell subset distribution towards a decreased frequency of regulatory T cells could potentially contribute to ongoing neurodegeneration in glaucoma, as suggested by Yang et al. (2019); DeMaio et al. (2022). These findings on immune functions align with previous results and underscore the significant role of the immune system in the pathogenesis of glaucoma. While no specific studies had directly investigated differences in non-immune-related gene expression profiles between high- and low-risk groups of glaucoma patients, existing research suggests potential variations in certain non-immune-related biological processes. For example, osteobridging protein (*OPN*) research has shown that *OPN* can facilitate cell adhesion, migration, and survival in human optic nerve head astrocytes by interacting with cell surface receptors like Integrin αV, Integrin β3, Integrin β5, and the non-integrin receptor *CD44*. These findings imply that *OPN* may play a significant neuroprotective role in glaucoma development (Neumann et al., 2014). Additionally, research on oligodendrocytes (OLs) in central nervous system (CNS) pathology has revealed a disease-related transcriptional program that could be influenced by neuroinflammation, consistent across various non-immune glial cells (Nat Neurosci, 2022). This shared transcriptional program suggests a similarity in non-immune-related gene expression profiles between high- and low-risk groups of glaucoma patients, particularly in genes related to neuroinflammation and cellular stress responses.

Additionally, the DGIdb database was utilized to predict small-molecule drugs that target the immune system for the treatment of POAG, laying a strong foundation for addressing this condition. Future research can focus on understanding the intricate mechanisms that control the expression of various immune components and their functions in the progression of POAG. These efforts would aid in the development of therapeutic strategies that target the immune system.

The study on identifying immune-related biomarkers for glaucoma through gene expression profiling acknowledges its innovative approach but also presents inherent limitations that warrant further discussion for a comprehensive understanding. A more detailed exploration of the study’s limitations could enhance its contribution to the field by addressing potential biases in patient samples, variations in disease severity, and other factors that may impact the generalizability of the findings. Initially, the study’s reliance on specific datasets and the retrospective nature of the analysis may limit the diversity and representativeness of the patient samples. And despite the validation of the hub genes in cell models, supporting evidence is still lacking in recent sequencing data, specifically at the single-cell level. Interestingly, the RGC-5 cell line, initially thought to be derived from rats but later identified as mouse-origin (Van Bergen et al., 2009), has significantly impacted retinal ganglion cell (RGC) neurobiology and glaucoma research. Its suitability for high-throughput screening has enabled deep exploration of the pathways affecting RGC survival and degeneration, critical for developing glaucoma therapies (Yang and Sun, 2023). Furthermore, RGC-5 has been pivotal in studying neuroprotective strategies and testing potential pharmacological treatments, offering insights into glaucoma’s complex pathogenesis (Shen et al., 2021). Despite the need for careful interpretation of study results due to its mischaracterization, the RGC-5 cell line’s contribution to understanding glaucoma’s neurobiological aspects underscores the value of such models in disease research (Sippl and Tamm, 2014). Additionally, the study’s computational and bioinformatics methods, while powerful, may not fully capture the complexity of immune responses in glaucoma, suggesting a need for further validation through experimental models or clinical trials. The identification of immune-related genes (IRGs) as potential diagnostic markers is promising, yet the study could benefit from a deeper investigation into the mechanisms by which these IRGs influence glaucoma pathogenesis and progression. Addressing these limitations through expanded datasets, prospective studies, and multidisciplinary research could pave the way for more personalized and effective diagnostic and therapeutic strategies for glaucoma.

## Conclusion

In conclusion, the IRGs signature proposed in this study can have a substantial impact on determining clinical outcomes for POAG patients. Conducting a comprehensive investigation of the immune response in POAG will contribute to a better understanding of the disease’s pathogenesis and its potential as a biomarker and treatment target. This, in turn, will facilitate the development of more effective treatment strategies.

## Data Availability

The datasets presented in this study can be found in online repositories. The names of the repository/repositories and accession number(s) can be found in the article/Supplementary Material.
