# Supplementary material for: Identification of immune-related biomarkers for glaucoma using gene expression profiling
Source: Front Genet. 2024 Apr 17;15:1366453. doi: 10.3389/fgene.2024.1366453 (PMC11062407; doi:10.3389/fgene.2024.1366453)
Supplement: Supplementary file 1 [file Table1.docx]

Supplementary Table S1

Clinical characteristics of the glaucoma in high-risk group and in low-risk group.

| Characteristic | High risk (n = 13) | Low risk (n = 15) | *p*-Value |
| --- | --- | --- | --- |
| Gender (F/M) | 6/7 | 8/7 | 0.710 ^N.S^ |
| Age (years) | 64.38±15.108 | 61.87±7.652 | 0.406 ^N.S^ |
| Angle type(O/C) | 6/7 | 3/12 | 0.011* |
| BCVA | 0.28±0.193 | 0.57±0.310 | 0.008** |
| Intraocular pressure(mmHg) | 28.92±13.269 | 20.53±5.902 | 0.065 ^N.S^ |
| Optic atrophy, n (%) | 13(100) | 7(47) | 0.002** |
| Visual field defect(-dB) | 26.54±6.375 | 3.05±1.484 | 0.000*** |
| Average RNFL thickness(mm) | 65.00(57.00, 69.00) | 90.50(83.25,102.50) | 0.001** |
| Central corneal thickness(µm) | 520.91±25.86 | 531.82±24.85 | 0.325 ^N.S^ |
| Anterior chamber depth(mm) | 2.59±0.28 | 2.29±0.25 | 0.021* |
| Lens thickness(mm) | 4.77±0.41 | 5.00±0.34 | 0.220 ^N.S^ |
| Axial length(mm) | 22.86±0.95 | 22.66±0.85 | 0.609 ^N.S^ |
| SBP(mmHg) | 118.30±10.48 | 130.73±12.92 | 0.027* |
| DBP(mmHg) | 73.64±10.86 | 84.92±6.04 | 0.005** |
| MAP(mmHg) | 87.23±8.92 | 100.18±7.25 | 0.002** |
| WBC (10^9^/L) | 5.46±1.52 | 6.34±1.19 | 0.149 ^N.S^ |
| Lymphocyte count(10^9^/L) | 1.80±0.31 | 1.49±0.30 | 0.033* |
| Monocyte count(10^9^/L) | 0.39±0.12 | 0.41±0.13 | 0.743 ^N.S^ |
| Neutrophil cell count(10^9^/L) | 3.19±1.24 | 4.20±1.06 | 0.015* |
| ALT (U/L) | 22.18±13.04 | 23.58±12.56 | 0.796 ^N.S^ |
| AST (U/L) | 23.82±5.81 | 22.67±5.57 | 0.632 ^N.S^ |
| TBIL (μmol/L) | 8.00±3.65 | 8.85±2.12 | 0.514 ^N.S^ |
| DBIL (μmol/L) | 3.51±1.30 | 3.63±1.03 | 0.815 ^N.S^ |
| γ-GGT (U/L) | 24.36±10.89 | 28.92±24.71 | 0.580 ^N.S^ |
| Creatinine (mmol/L) | 76.00±16.35 | 67.60±14.50 | 0.230 ^N.S^ |
| Urea (mmol/L) | 5.93±1.54 | 5.58±1.55 | 0.600 ^N.S^ |

N.S not significant, * *p* < 0.05, ** *p* < 0.01, *** *p* < 0.001. CON, healthy controls; POAG, primary open angle glaucoma; PACG, primary angle closure glaucoma; F, female; M, male; BMI, body mass index; BCVA, best corrected visual acuity; SBP, systolic blood pressure; DBP, diastolic blood pressure; MAP, mean arterial pressure; BCVA, best corrected visual acuity; WBC, white blood cell; ALT, alanine aminotransferase; AST, aspartate aminotransferase; TBIL, total bilirubin; DBIL, direct bilirubin; γ-GGT, gamma-glutamyltransferase.
