# Supplementary material for: Identification of immune-related biomarkers for glaucoma using gene expression profiling
Source: Front Genet. 2024 Apr 17;15:1366453. doi: 10.3389/fgene.2024.1366453 (PMC11062407; doi:10.3389/fgene.2024.1366453)
Supplement: Supplementary file 2 [file Table2.docx]

Supplementary Table S2

The sequences of the primers for qPCR.

| Genes | Forward | Reverse |
| --- | --- | --- |
| CD40LG | GTCTGTTCACTTGGGCGGAG | TCACTTGGCTTGCTTCAGTCA |
| TEK | TCGCAGGAGAACTGGAGGTT | ATGGCAAAAGCAGGGTCTGT |
| MDK | GCACTGGTAAAACCGAACTCC | CAGGTCCACTCCGAACACTC |
| β-actin | CGAGGCCCAGAGCAAGAGAG | CGGTTGGCCTTAGGGTTCAG |
