# Supplementary material for: Identification of immune-related biomarkers for glaucoma using gene expression profiling
Source: Front Genet. 2024 Apr 17;15:1366453. doi: 10.3389/fgene.2024.1366453 (PMC11062407; doi:10.3389/fgene.2024.1366453)
Supplement: Supplementary file 3 [file Table3.docx]

| Supplementary Table S3  Drug Gene Interaction Database (DGIdb) was used to predict drugs targeting marker genes. | | | | | | | | |
| --- | --- | --- | --- | --- | --- | --- | --- | --- |
| Gene Symbol | Drug | Mode of administration | Study population/model | | | Results | Target/Mechanism | References |
|  |  |  | In vitro | In vivo | Clinical trial |  |  |  |
| **CD40LG** | Atorvastatin | Oral |  |  | Association of statin agent with risk of open-angle glaucoma | Associating with a reduction in OAG risk |  | PMID:  28114645 |
|  |  | Intraperitoneal injection |  | Rat model |  | Exerting neuroprotective effects | Promoting the survival of RGCs and suppressing chronic OHT-mediated glial activation in the retina | PMID:  34830387 |
|  |  | Gavage; In vitro treatment | Primary human trabecular meshwork cells(HTM) | Mice model |  | Reducing IOP in ocular hypertension and suppressing ECM in trabecular meshwork | Reducing TGF-β2-induced ocular hypertension | PMID:  35417030 |
|  | Rosiglitazone | Oral; In vitro treatment | Primary mouse cortical astrocytes | Mice model |  | Stimulating Ucp2 expression in RGCs | PPAR-γ-dependent transcriptional activator of Ucp2 | PMID:  30906248 |
|  |  | Eye drops; In vitro treatment | Human Tenon fibroblasts(HTF) | Rabbit model |  | Inhibiting the progression of pro-fibrotic | Peroxisome proliferator-activated receptor γ (PPARγ)-selective agonist | PMID: 31247081 |
|  | Fenofibrate |  |  |  |  |  |  |  |
|  | Toralizumab |  |  |  |  |  |  |  |
|  | PG-102 |  |  |  |  |  |  |  |
|  | Aldesleukin |  |  |  |  |  |  |  |
|  | Selicrelumab |  |  |  |  |  |  |  |
|  | Imatinib |  |  |  |  |  |  |  |
|  | Dapirolizumab pegol |  |  |  |  |  |  |  |
|  | Ruplizumab |  |  |  |  |  |  |  |
|  | Lucatumumab |  |  |  |  |  |  |  |
| **TEK** | Regorafenib | Eye drops |  | Beagle model |  | Maintaining bleb formation and reducing IOP | Multi-kinase inhibitor of fibroblast growth factor receptor (FGFR) | PMID: 31861830 |
|  | Razuprotafib | Eye drops |  |  | Drug: Latanoprost ophthalmic solution Drug: AKB-9778 4% Drug: Placebo ( NCT04405245) | Reducing IOP in patients with OAG/OHT as an adjunct to latanoprost | Inhibiting VE-PTP (vascular endothelial-protein tyrosine phosphatase) and enhancing Tie2 (tyrosine kinase with immunoglobulin-like and Epidermal Growth Factor (EGF)-like domains 2) activation and signaling. | PMID:  34989803 |
|  | Cabozantinib |  |  |  |  |  |  |  |
|  | MGCD-265 |  |  |  |  |  |  |  |
|  | Glesatinib |  |  |  |  |  |  |  |
|  | GW559768X |  |  |  |  |  |  |  |
|  | Vandetanib |  |  |  |  |  |  |  |
|  | Pexmetinib |  |  |  |  |  |  |  |
|  | Foretinib |  |  |  |  |  |  |  |
|  | Loperamide |  |  |  |  |  |  |  |
|  | Linifanib |  |  |  |  |  |  |  |
|  | Rebastinib |  |  |  |  |  |  |  |
|  | Ampicillin |  |  |  |  |  |  |  |
|  | Cetirizine |  |  |  |  |  |  |  |
|  | Altiratinib |  |  |  |  |  |  |  |
|  | CE-245677 |  |  |  |  |  |  |  |
|  | CEP-11981 |  |  |  |  |  |  |  |
| **MDK** | Tretinoin |  |  |  |  |  |  |  |
