# Supplementary material for: Identification of immune-related biomarkers for glaucoma using gene expression profiling
Source: Front Genet. 2024 Apr 17;15:1366453. doi: 10.3389/fgene.2024.1366453 (PMC11062407; doi:10.3389/fgene.2024.1366453)
Supplement: Supplementary file 4 [file Image1.pdf]

Fig. S1

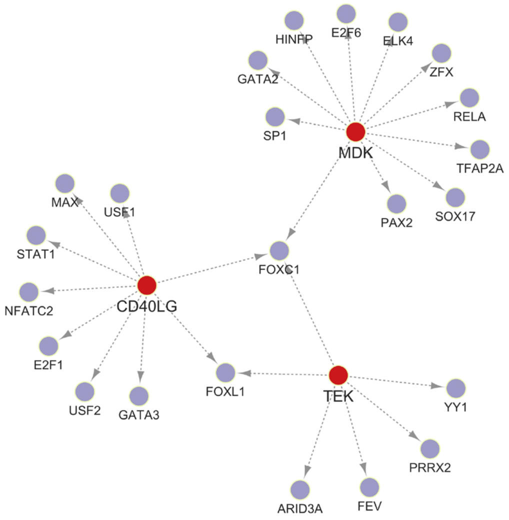

Fig. S1 A comprehensive transcriptional regulatory network of key immune-related genes (*CD40LG*, *MDK* and *TEK*) was constructed by visualization through Cytoscape. CD40 Ligand, *CD40LG*; Midkine, *MDK*; and *TEK* Receptor Tyrosine Kinase, *TEK*.

Fig. S2

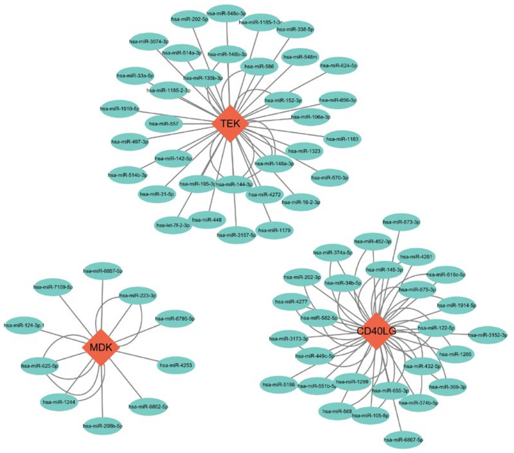

Fig. S2 The three key genes through the miRWalk database to obtain their possible miRNAs. CD40 Ligand, *CD40LG*; Midkine, *MDK*; and *TEK* Receptor Tyrosine Kinase, *TEK*.

Fig. S3

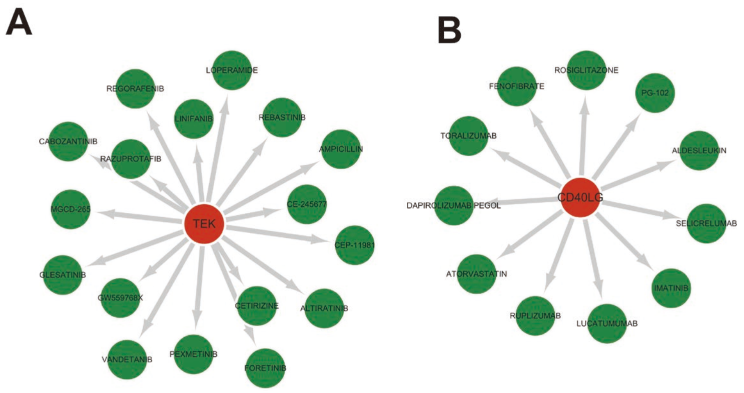

Fig. S3 The gene-drug network. The drug-gene network of *TEK* **A** and *CD40LG* **B.**

Fig. S4

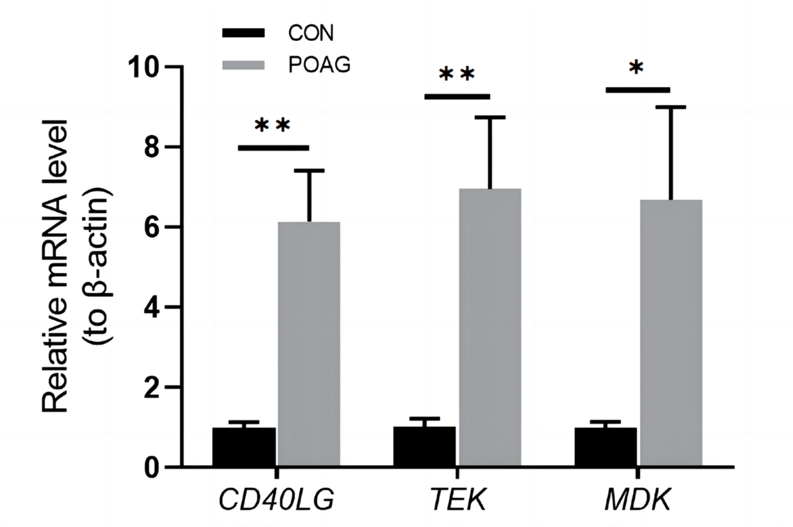

Fig. S4 RT-qPCR to detect mRNA expression levels of *CD40LG*, *TEK*, *MDK* in NMDA-treated RGCs. $^{*}P$<0.05, $^{**}P$<0.01, $^{***}P$<0.001. RT-qPCR: Real-time reverse transcriptase-polymerase chain reaction; NMDA, N-methyl-d-aspartic acid; RGCs, retinal ganglion cell line; POAG, RGCs with NMDA treatment; CON, RGCs without drug treatment.
